# Machine Learning in Prediction of Bladder Cancer on Clinical Laboratory Data

**DOI:** 10.3390/diagnostics12010203

**Published:** 2022-01-14

**Authors:** I-Jung Tsai, Wen-Chi Shen, Chia-Ling Lee, Horng-Dar Wang, Ching-Yu Lin

**Affiliations:** 1 Ph.D. Program in Medical Biotechnology, College of Medical Science and Technology, Taipei Medical University, Taipei 11031, Taiwan; d609108005@tmu.edu.tw; 2 Institute of Biotechnology, National Tsing Hua University, Hsinchu 30013, Taiwan; tt22407@gmail.com; 3 Pathology Department, MacKay Memorial Hospital, Taipei 10449, Taiwan; g660108001@tmu.edu.tw; 4 Department of Life Science, National Tsing Hua University, Hsinchu 30013, Taiwan; 5 Institute of Systems Neuroscience, National Tsing Hua University, Hsinchu 30013, Taiwan; 6 School of Medical Laboratory Science and Biotechnology, College of Medical Science and Technology, Taipei Medical University, Taipei 11031, Taiwan

**Keywords:** machine learning, bladder cancer, feature selection, clinical laboratory data

## Abstract

Bladder cancer has been increasing globally. Urinary cytology is considered a major screening method for bladder cancer, but it has poor sensitivity. This study aimed to utilize clinical laboratory data and machine learning methods to build predictive models of bladder cancer. A total of 1336 patients with cystitis, bladder cancer, kidney cancer, uterus cancer, and prostate cancer were enrolled in this study. Two-step feature selection combined with WEKA and forward selection was performed. Furthermore, five machine learning models, including decision tree, random forest, support vector machine, extreme gradient boosting (XGBoost), and light gradient boosting machine (GBM) were applied. Features, including calcium, alkaline phosphatase (ALP), albumin, urine ketone, urine occult blood, creatinine, alanine aminotransferase (ALT), and diabetes were selected. The lightGBM model obtained an accuracy of 84.8% to 86.9%, a sensitivity 84% to 87.8%, a specificity of 82.9% to 86.7%, and an area under the curve (AUC) of 0.88 to 0.92 in discriminating bladder cancer from cystitis and other cancers. Our study provides a demonstration of utilizing clinical laboratory data to predict bladder cancer.

## 1. Introduction

Bladder cancer has been noted as the 10th most common cancer in the world [1]. The incidence of bladder cancer is rising globally, especially in developed countries, such as U.S.A, Germany, and Taiwan; according to GLOBOCAN, 573,278 new cases of bladder cancer and 212,536 new deaths [2]. Furthermore, bladder cancer is observed in men more than in women, with respective incidence and mortality rates of 9.5 and 3.3 per 100,000 among men, which are four times those among women globally [2]. Moreover, smoking is considered the major risk factor in patients with bladder cancer [3]. The gold standard procedure for diagnosing bladder cancer is cystoscopy, with a sensitivity 88–100% and specificity 77.1–97% [4]. Currently, urinary cytology is considered a major non-invasive method to diagnose bladder cancer with high specificity, but only 38% sensitivity [5]. Therefore, a screening method with high sensitivity and high specificity is urgently needed for the diagnosis of bladder cancer.

Clinical chemistry tests and urinalysis are the major diagnostic screening test in the clinical laboratory [6]. The alteration of each test can be interpreted as a relationship with diseases; for instance, aspartate aminotransferase (AST) and alanine aminotransferase (ALT) are the enzymes from liver, pancreas, and kidneys [7]. Furthermore, an increased level of AST and ALT is used in the diagnosis of liver disease; however, it is also related to the damage of other organs [7]. Large amounts of alkaline phosphatase (ALP) can be found in liver, bone, intestine, and placenta; while, ALP can be considered an indicator of bone formation [8]. In addition, the analysis of isoenzymes of ALP is an aid in diagnosing bone and liver disease [9]. Studies indicated that ALP may be related to diseases such as chronic kidney disease [10], rheumatoid disease [11], malignant disease [12], and prostate cancer [13]. The physiological meaning of the alteration in ions, such as potassium ions, sodium ions, calcium ions, and chloride, is complicated. Studies indicated that the alteration of sodium ions and calcium ions may be related to hypertension [14], alkalosis [15], cancers [16], and multiple sclerosis [17]. The serum level of albumin can be considered the sum of synthesis, degradation, and distribution [18]. In addition, albumin in the blood exhibits many biological functions, including transport of endogenous and exogenous compounds, modulation of capillary permeability, neutrophil homeostasis, and free radical scavenging [19]. The alteration of serum albumin level has been found to be correlated with coronary heart disease [20], bowel disease [21], and liver disease [19]. Whereas the urine albumin has been reported to be associated with chronic kidney disease [22] and heart disease [23]. Creatinine is the amino acid compound from creatine and is released into the blood mainly from muscles; meanwhile, the creatinine in the blood is released constantly and filtered by glomerulus in the kidney [24]. Furthermore, the creatinine in blood can be used to calculate the glomerular filtration rate, to evaluate the function of the kidney [25]. Therefore, the alteration of creatinine in the blood may be related to muscle disease and kidney disease [26]. A complete urinalysis consists of color, clarity, specific gravity, and chemical analyses, as well as examination of urine sediment [27]. A chemical analysis is usually conducted to examine pH, glucose, ketones, occult blood, bilirubin, and protein with dipstrip systems [28]. Aberrant results in chemical analysis can occur in certain diseases; for instance, ketonuria can be found in the urine from patients with diabetes; while, hematuria indicates bleeding within the urinary tract [29]. Moreover, the examination of urine sediment may be found with crystal, erythrocytes, leukocytes, bacteria, and casts, which provide information about the urinary tract system [30]. However, interpreting clinical tests individually may cause a misleading diagnosis [31]. The laboratory test results can be interpreted with experienced clinicians, but it can also be integrated and interpreted with artificial intelligence (AI), such as machine learning algorithms [32].

Machine learning is a type of AI whereby computers independently learn from data, without human intervention [33]. Furthermore, machine learning has different methods to approach the learning process [34]. Various machine learning algorithms have been frequently used in medical studies. Tree based models, such as decision tree, random forest, extreme gradient boosting (XGBoost), and light gradient boosting machine (GBM) have been frequently used in medical studies [35,36,37]. Moreover, support vector machine has been considered an alternative approach for managing clinical data [38]. Recently, many studies in machine learning applications have been introduced into clinical practice. Machine learning has become a powerful tool for improving diagnostic and prognostic accuracy in cancer, with various kinds of data; for instance, Cai et al. exploited genomic data to create a panel of 16 DNA methylation markers and combined them with random forest to provide a classification power with an accuracy of 86.54% in classifying lung cancer [39]. Routine clinical and laboratory data were used to establish machine learning models to identify lung cancer in an early stage [40]. An elegant study from Obaid et al. demonstrated the potential of integrating image data with machine learning to diagnose breast cancer and received an accuracy of 98.1% [41]. As for bladder cancer, Garapati et al. built an objective computer-aided system to identify the stage of bladder cancer with CT urography [42]. Additionally, machine learning was also applied to metabolomics to recognize early and late stages of bladder cancer [43]. However, we found that no study has applied machine learning with clinical laboratory data for improving the diagnostic accuracy of patients with bladder cancer.

Herein, we collected clinical laboratory data, including biochemistry tests and urinalysis from 1336 patients with cystitis, bladder cancer, and other types of cancer in Mackay Memorial Hospital. We combined sampling techniques and two-step feature selection to exploit the clinical laboratory dataset. Furthermore, five different machine learning models were trained and validated with the selected dataset. Moreover, the accuracy, precision, f1 score, sensitivity, specificity, and area under the area of receiving operating characteristic curve (AUC) were calculated to evaluate the model performance.

## 2. Materials and Methods

### 2.1. Patient Cohort

We collected clinical laboratory test data from 144 patients with cystitis (56 female and 88 male patients, aged 60.12 ± 11.99), 200 patients with kidney cancer (62 female and 138 male patients, aged 63.41 ± 10.45), 201 patients with prostate cancer (201 male patients, aged 71.83 ± 6.42), 591 patients with bladder cancer (205 female and 386 male patients, aged 66.73 ± 9.4), and 200 patients with uterus cancer (200 female patients, aged 60.86 ± 10.26) in Mackay Memorial Hospital from January 2017 to February 2020. Patients with cancers were diagnosed by clinician and confirmed via pathological report. MacKay Memorial Hospital Institutional Review Board approved the study protocol (20MMHIS200e (8 July 2020)).

### 2.2. Statistical Analysis

Description analyses were performed with SPSS 19.0 (IBM, Chicago, IL, USA). Continuous variables are presented as mean ± SD or median (25th and 75th percentile). The categorical variables are presented as number and percentage. The clinical laboratory test and characteristics were compared with a *t*-test or Mann–Whitney U test for continuous variables and chi-square test for categorical variables.

### 2.3. Data Processing

We collected clinical laboratory data with 56 laboratory tests results. The laboratory test results with more than 50% of missing data were removed. After that, we received 31 laboratory test results with missing rate, varying between 0 to 44.1% (Table 1). The missing data was filled with the mean value for continuous value and median value for categorical value from each feature in the whole data. Features that were missing in the data in certain classifications were avoided in the feature selecting, model training, and validating. For instance, A/G ratio and urine epithelium was not included while discriminating cystitis from other cancers. The oversampling and undersampling techniques from imblearn v0.0 package were used for the problem of imbalanced data [44].

### 2.4. Feature Selection and Machine Learning

We used an InfoGainAttributeEval (InfoGain) + Ranker method with default parameters to perform feature selection with WEKA (vers. 3.8.3) (Table 2). Furthermore, optimized models were used to conduct a forward selection, as mentioned in a previous study [45]. The models we built in this study were based on decision tree (DT), random forest (RF), support vector machine (SVM), XGBoost, and lightGBM, with 10-fold cross validation with scikit-learn (vers. 0.21.3). The parameters were tuned before the experiments. For DT, the initial value of tree depth was set from 1–10, with a step of 1. The kernel of the model was set to entropy or gini. For RF, the initial value of the tree number was set at 100 and increased by 100 until 500. The kernel of model was set to gini or entropy. For SVM, the initial value of gamma was set from 10-6 to 10-10, with a step of 0.1. The initial value of C was set from 10-6 to 10-7 with a step of 10. The kernel of SVM was set to RBF. For XGBoost, the initial value of eta was from 0.01 to 0.2, with a step of 0.05. The initial value of depth was set from 1 to 10, with a step of 1. As for lightGBM, the initial value of leaves was set from 50 to 400, with a step of 50. The initial value of depth was set from 1 to 10, with a step of 1. The parameters of the machine learning were tuned via training and validating with the whole dataset. The parameters that obtained the highest accuracy were selected (Table 2). A confusion matrix was used in this study to calculate the accuracy, precision, sensitivity, specificity, and f1 score (Table 3). The value of AUC was calculated from scikit-learn (vers. 0.21.3).

(1) $$\mathrm{Accuracy}=\frac{\mathrm{TP}+\mathrm{TN}}{\mathrm{TP}+\mathrm{FP}+\mathrm{TN}+\mathrm{FN}}$$

(2) $$\mathrm{Precision}=\frac{\mathrm{TP}}{\mathrm{TP}+\mathrm{FP}}$$

(3) $$\mathrm{Sensitivity}=\frac{\mathrm{TP}}{\mathrm{TP}+\mathrm{FN}}$$

(4) $$\mathrm{Specificity}=\frac{\mathrm{TN}}{\mathrm{FP}+\mathrm{TN}}$$

(5) $$f1\;\mathrm{score}=\frac{2}{\frac{1}{\mathrm{precision}}+\frac{1}{\mathrm{sensitivity}}}$$

## 3. Results

### 3.1. Clinical Characterisitcs and Clincal Laboratory Data from Patients

The differences in demographics, baseline characteristics, and laboratory data between healthy groups and other cancers are summarized in Table 4. We found that albumin, ALP, BUN, chloride, creatinine, direct bilirubin, eGFR, pH, potassium, total protein, nitrite, strip WBC, and urine occult blood were significantly different in patients with kidney cancer compared to patients with cystitis. Furthermore, we discovered that ALP, AST, BUN, calcium, creatinine, sodium, urine epithelium counts, and urine occult blood had significant differences in patients with prostate cancer compared to patients with cystitis. As for bladder cancer, the statistical results shown that ALP, BUN, calcium, chloride, creatinine, direct bilirubin, eGFR, glucose, specific gravity, total protein, and uric acid were significantly different compared to patients with cystitis. Lastly, ALP, BUN, calcium, chloride, creatinine, eGFR, glucose, potassium, sodium, urine epithelium count, urine protein, urobilinogen, and urine occult blood were significantly different between patients with uterus cancer and patients with cystitis.

### 3.2. Feature Selection and Sampling Technique Experiment

**Figure 1:**
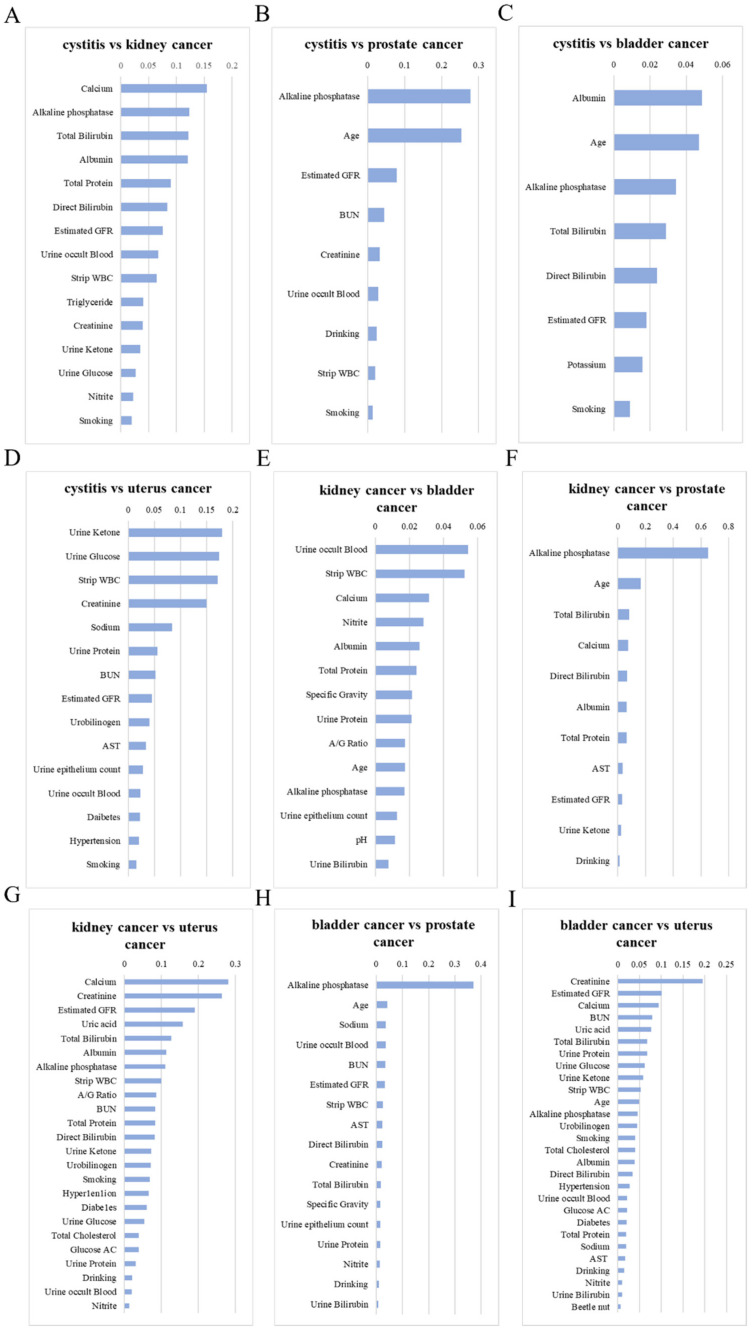
The feature selection results of (**A**) cystitis vs. kidney cancer, (**B**) cystitis vs. prostate cancer, (**C**) cystitis vs. bladder cancer, (**D**) cystitis vs. uterus cancer, (**E**) kidney cancer vs. bladder cancer, (**F**) kidney cancer vs. prostate cancer, (**G**) kidney cancer vs. uterus cancer, (**H**) bladder cancer vs. prostate cancer, and (**I**) bladder cancer vs. uterus cancer.

**Figure 2:**
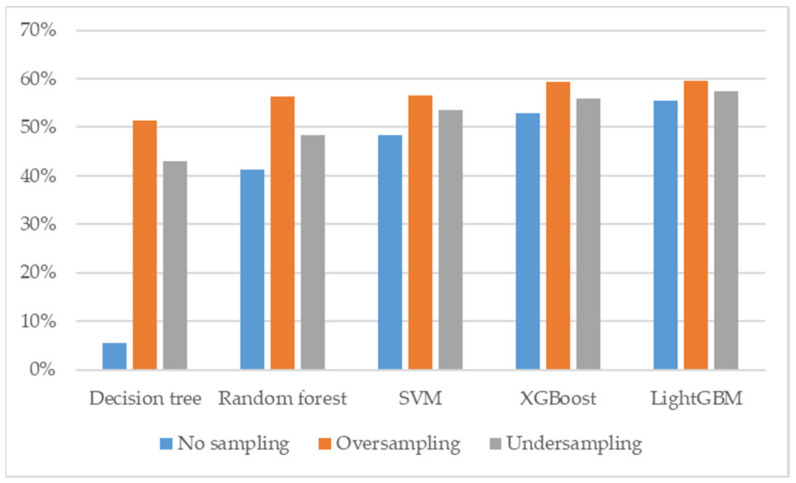
The specificity comparison of sampling techniques in five different models.

To reduce the noise in the dataset, we used InfoGain + Ranker to rank the features between groups (Figure 1). The top feature in each group was assembled as a set of selected features, including calcium, alkaline phosphate, albumin, urine ketone, urine occult blood, and creatinine (Table 5). We used the dataset to train and validate five models, including decision tree, random forest, SVM, XGBoost, and lightGBM. However, the evaluation parameter may not reflect the learning results, due to imbalanced data from bladder cancer compared to other groups. We used the python package named imbalanced-learn to solve the sample imbalance issue. Five models without any sampling techniques were trained and validated with the dataset. In differentiating patients with bladder cancer from patients with cystitis, the models received an accuracy 77.2–78.8%, precision 76.2–80.8%, f1 score 76.6–86.9%, sensitivity 77.7–95.8%, specificity 5–55.4%, and roc 0.592–0.729 (Table S1). After the oversampling technique was applied in the training and validating, the accuracy was adjusted to 73.4–78.8%, precision was adjusted to 74.9–81%, f1 score was adjusted to 75.6–81.4%, sensitivity was adjusted to 78–84.3%, and specificity was adjusted to 51.3–59.3% (Table S1 and Figure 2). An undersampling technique was also tested in our study. The accuracy was adjusted to 76.3–78.3%, precision was adjusted to 78.6–80.6%, f1 score was adjusted to 77.8–80.3%, sensitivity was adjusted to 79.0–83.9%, specificity was adjusted to 42.9–57.4%, and the roc was adjusted to 0.69–0.74 (Table S1). To further optimize our models, we conducted forward selection with the sampling technique in five different models.

### 3.3. Model Evaluation and Comparison 

**Figure 3:**
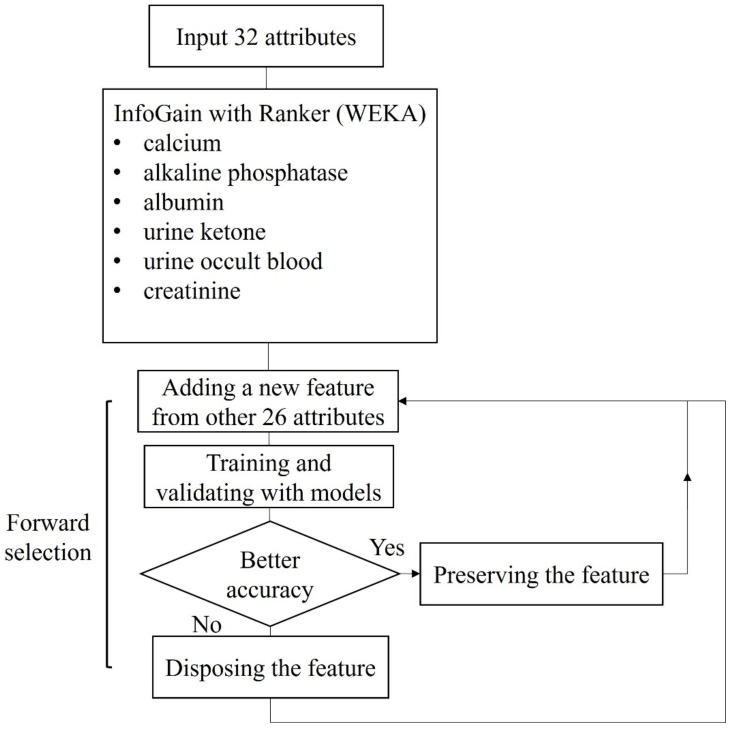
The block diagram of the forward selection method.

The forward selection method is illustrated in Figure 3. The features from forward selection may be different, due to the models and the classes in the dataset. In the forward selection experiment, we focused on discriminating the patients with cystitis and patients with bladder cancer. In the results of the decision tree classifier, the features including ALT, AST, potassium, sodium, specific gravity, strip WBC, total protein, triglyceride, urine epithelium count, and uric acid were further selected. The decision tree classifier was trained and validated with features from WEKA and forward selection. The model received an accuracy of 76.2%, a precision of 77.9%, a f1 score of 74.6%, a sensitivity of 73.2%, a specificity of 78.1%, and an AUC of 0.77 in differentiating patients with bladder cancer from patients with cystitis (Table 6). In the results of the random forest classifier, the feature including ALT was selected. The random forest classifier was trained and validated with features from WEKA and forward selection. The model received an accuracy of 83.1%, a precision of 78.2%, a f1 score of 81.6%, a sensitivity of 85.5%, a specificity of 79.4%, and an AUC of 0.88 in discriminating patients with bladder cancer from patients with cystitis (Table 6). In the results of SVM, features including ALT, BUN, chloride, direct bilirubin, nitrite, and pH were further selected. The SVM was trained and validated with features from WEKA and forward selection. The model received an accuracy of 71.7%, a precision of 81.9%, a f1 score of 65.5%, a sensitivity of 55.7%, a specificity of 86.7%, and an AUC of 0.73 in identifying patients with bladder cancer and patients with cystitis (Table 6). In the results of XGBoost, features including ALT, AST, BUN, chloride, direct bilirubin, pH, potassium, sodium, total bilirubin, and total cholesterol were further selected (Table 6). The XGBoost model was trained and validated with features from WEKA and forward selection. The model received an accuracy of 82.8%, a precision of 84.7%, a f1 score of 82.7%, a sensitivity of 81.4%, a specificity of 83.3%, and an AUC of 0.87 in discriminating patients with bladder cancer from patients with cystitis (Table 6). In the results of lightGBM, features including ALT, and diabetes were further selected. The lightGBM model was trained with features from WEKA and forward selection. The model received an accuracy of 87.6%, a precision of 86.3%, a f1 score of 87.7%, a sensitivity of 89.5%, a specificity of 85.5%, and an AUC of 0.93 in identifying patients with bladder cancer and patients with cystitis (Table 6). 

**Figure 4:**
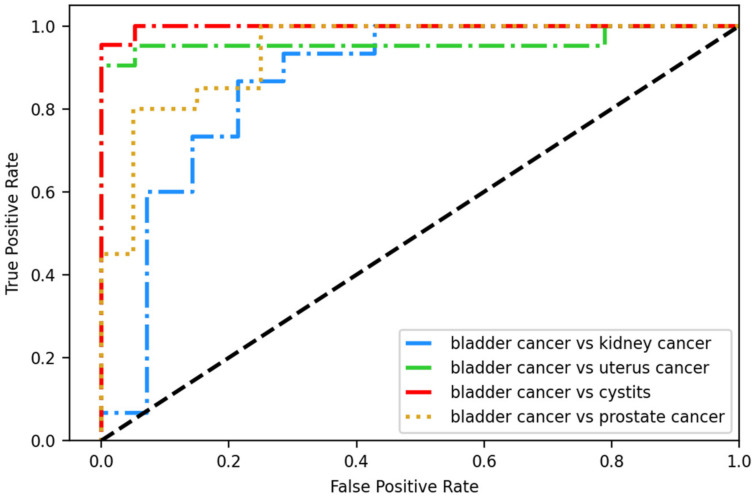
The receiver operating characteristic curve (ROC) plot of separating bladder cancer from cystitis or other cancers.

The lightGBM with selected features received the highest score for accuracy, precision, f1 score, sensitivity, and AUC. Therefore, we further evaluated the model performance in differentiating bladder cancer from other cancers. The model received an accuracy of 84.8% to 86.9%, a precision of 83% to 87.1%, a f1 score of 84.5% to 87.7%, a sensitivity of 84.4% to 87.8%, a specificity of 82.9% to 86.7%, and an AUC of 0.88 to 0.92 (Table 7 and Figure 4).

## 4. Discussion

Machine learning has been significantly developed in the past decade. Many machine learning applications have been created with different types of data, including genomic data, transcriptomic data, proteomic data, image data, electronic health records (EHR), and clinical laboratory data [46,47,48]. However, the most intriguing question is whether machine learning can be applied to medical diagnosis [49]. Obermeyer et al. suggested that machine learning applied to clinical laboratory data can dramatically improve prognosis and diagnostic accuracy [50]. Moreover, compared to novel biomarkers, a decision-making assist program based on clinical laboratory data can be considered a fast and cheap solution for improving the accuracy of diagnosis.

Herein, we utilized the clinical laboratory dataset coupled with machine learning algorithms to discriminate patients with bladder cancer from patients with cystitis. Missing values and imbalanced data were the two major challenges we encountered in this study. Missing values can be categorized as missing complete at random (MCAR), missing at random (MAR), and missing not at random (MNAR) [51]. Several methods can solve this issue, such as collecting more samples, removing the subjects with missing values, filling with mean or median, and imputing missing values [52,53]. Multiple imputation (MI) is considered a good method to calculate missing values from existing data [54]. An elegant study from Hong et al. performed MI with models such as random forest and received a good accuracy [55]. However, in our experiment, MI did not receive applicable results (data not shown). We speculated that MI needs a larger sample size or strongly correlated features to obtain enough characteristics from the existing data. The skewed data distribution of one class over another is considered imbalanced data [56]. The imbalanced data causes classification problems during the training of machine learning algorithms [57]. Therefore, we used sampling techniques, including oversampling and undersampling, to reduce the error. The study performed by Mohammed et al. suggested that oversampling has a better performance with certain classifiers and evaluation metrics [58]. The oversampling method was applied to molecular description data by Chang et al., and reported that it could be used to reduce the overfitting problem [59]. However, oversampling has some disadvantages, such as sample overlapping, noise interference, and blindness of neighbor selection [60]. The main disadvantage of oversampling is that by making copies from existing data, overfitting is likely; in contrast, the main disadvantage of undersampling is the discarding of potentially useful data [61]. Instead of acquiring the highest performance from the models, our goal was to achieve an authentic performance from the models with our dataset. Furthermore, when faced with imbalanced data, it requires more than a one-step solution to improve the accuracy of the model [62]. Thus, in our experiment, we applied tools including undersampling techniques, feature selection, and improved models to increase the diagnostic accuracy in bladder cancer.

From our two-step feature selection, calcium, ALP, albumin, urine ketone, urine occult blood, and ALT were selected from the clinical laboratory data. Michel et al. reported that hypercalcemia was only observed in several patients with bladder cancer. Furthermore, the data shown that the hypercalcemia was caused by increasing levels from the tumor [63]. Moreover, Rosa et al. reported that an increasing level of calcium is common in various cancers, but rare in bladder cancer [64]. In addition, Huang et al. suggested that the elevation of calcium in blood may be considered as an indicator of bone metastasis in bladder cancer [65]. These studies suggested that calcium is a good feature for discriminating bladder cancer from other cancers. ALP has been considered a prognostic biomarker in patients with prostate cancer [66]. Therefore, ALP was selected as the top feature for prostate cancer versus cystitis or other cancers in our study. Furthermore, Braendengen et al. reported that an increased level of ALP in serum did not improve the accuracy of a bone scan used for evaluation precystectomy, which suggested a low correlation between ALP and bladder cancer [67]. These studies indicated that ALP can be used to identify prostate cancer from other cancers without interfering with the classification of bladder cancer. Albumin and globulin play an important role in immunity and inflammation; therefore, several studies have been proposed the ratio of albumin to globulin ratio as a biomarker in gastric cancer and lung cancer [68]. In addition, Quhal et al. reviewed the albumin to globulin ratio in 1096 patients with non-muscle-invasive bladder cancer and found that the ratios independently predicted the progression of disease [69]. Moreover, Tan et al. proposed that the ratio of albumin to ALP can be used as a prognostic biomarker in upper tract urothelial carcinoma [70]. Urine ketone is one of the routine urinalysis. Only a few studies reported an alteration of ketone in patients with bladder cancer in a metabolomics study [71]. However, ketone body in urine has been considered as a high correlation to diabetes [72]. Furthermore, the ketones in the blood and urine may indicate that the patients were suffering from diabetic ketoacidosis [73]. Moreover, a comprehensive systemic review suggested that diabetes mellitus was associated with bladder cancer [74]. These studies indicate that urine ketone is related to bladder cancer. Urine occult blood has been considered a screening indicator for bladder cancer [75]. Furthermore, a test for microhematuria found a strong correlation with bladder cancer in 46,842 patients [76]. However, urine occult blood can also be observed in other types of cancer, such as kidney cancer [77] or simply in a benign disease [78]. The lightGBM model we built in this study can discriminate bladder cancer from kidney cancer or cystitis with accuracies of 0.876 and 0.845. The ratio of AST and ALT was proposed as an indicator of liver function [79]. Recently, the ratio of AST and ALT has been discovered to have an association with bladder cancer [80]. Furthermore, Ha et al. suggested that the ratio of AST and ALT may further serve as a prognosis indicator in bladder cancer [81].

In this study, we trained and validated models with selected data from WEKA. Furthermore, a forward selection was performed with five different models, to optimize the model performance. Among the models, the lightGBM model had the highest performance, including an accuracy of 0.87, a precision of 0.86, a f1 score of 0.87, a sensitivity of 0.89, and an AUC of 0.93 in separating patients with bladder cancer from patients with cystitis (Table 6). Many studies have aimed at improving the diagnostic accuracy in bladder cancer. Wang et al. utilized machine learning algorithms to improve the tumor marker-based screening for multiple cancers and yielded a sensitivity of 0.81 and a specificity of 0.64 [82]. Shao et al. applied ultra-performance liquid chromatography coupled with time-of-flight mass spectrometry to acquire metabolites profiles in 152 samples from patients with bladder cancer and hernia; furthermore, the decision tree model embedded in this study obtained an accuracy of 76.6%, a sensitivity of 71.88%, and a specificity of 86.67% [83]. Wittmann et al. developed a random forest model with a set of metabolites selected based on statistical significance, metabolic pathway coverage, and fold difference from global metabolomics profiling of urine. Moreover, the model was tested in two independent cohorts and received an AUC of 0.81 to 0.78 [84]. Belugina et al. developed a non-invasive potentiometric multisensory system to perform urine analysis. In addition, various models were used in this study and received an accuracy of 76%, a sensitivity of 80%, and a specificity of 75% [85]. Kouznetsova et al. used two modeling methods, including multilayer perceptron (MLP) and stochastic gradient descent (SGD) with logistic regression loss function to discriminate bladder cancer patients with metabolite profiling. The best performing model was able to identify bladder cancer patients with an accuracy of 82.54% [43]. Compared to those studies, the model we performed in this study provided a better sensitivity and specificity. For future work, we aim to collect data from different cohorts. Moreover, we are eager to build a model that can differentiate bladder cancer from cystitis and other cancers in our next work.

## 5. Conclusions

In summary, we used two-step feature selection to select eight clinical laboratory tests and established a prediction model for bladder cancer with lightGBM. Furthermore, sample techniques were also used in our study and adjusted the imbalanced data. Our study indicated the potential of utilizing clinical laboratory data to detect cancer.

## Figures and Tables

**Table 1:** The missing data rate in the clinical laboratory data.

Feature	Data Type	Missing Data	Missing Data (%)
A/G Ratio	Continuous	0.441	44.1
Albumin	Continuous	0.224	22.4
ALP	Continuous	0.378	37.8
ALT	Continuous	0.068	6.8
AST	Continuous	0.046	4.6
BUN	Continuous	0.018	1.8
Calcium	Continuous	0.428	42.8
Chloride	Continuous	0.084	8.4
Creatinine	Continuous	0.005	0.5
Direct Bilirubin	Continuous	0.303	30.3
Estimated GFR	Continuous	0.008	0.8
Glucose AC	Continuous	0.038	3.8
Nitrite	Categorical	0.026	2.6
Urine occult Blood	Categorical	0.026	2.6
pH	Continuous	0.026	2.6
Potassium	Continuous	0.035	3.5
Sodium	Continuous	0.037	3.7
Specific Gravity	Continuous	0.026	2.6
Strip WBC	Continuous	0.16	16
Total Bilirubin	Continuous	0.216	21.6
Total Cholesterol	Continuous	0.19	19
Total Protein	Continuous	0.285	28.5
Triglyceride	Continuous	0.204	20.4
Urine epitheilum (UL)	Continuous	0.43	43
Urine epithelium count	Continuous	0.02	2
Uric acid	Continuous	0.15	15
Urine Bilirubin	Categorical	0.026	2.6
Urine Glucose	Categorical	0.16	16
Urine Ketone	Categorical	0.16	16
Urine Protein	Categorical	0.026	2.6
Urobilinogen	Categorical	0.026	2.6

A/G Ratio: albumin globulin ratio. BUN: blood urea nitrogen.

**Table 2:** The parameters of machine learning and feature selection.

Algorithm Name	Parameter Name	Parameter Value
InfoGainAttributeEval	binarizeNumericAttributes	False
	doNotCheckCapabilities	False
	missingMerge	True
Ranker	generateRanking	True
	numToSelect	−1
Decision tree	criterion of tree	gini
	depth of tree	4
Random forest	criterion of tree	gini
	estimators	300
SVM	kernel	rbf
	C value	1000
	gamma	0.000001
XGBoost	eta	0.2
	depth of tree	7
LightGBM	number of leaf	100
	depth of tree	1

**Table 3:** The confusion matrix for evaluation of model performance.

		Patients	
		Bladder Cancer	Cystitis
**Prediction**	bladder cancer	true positive, TP	false positive, FP
	cystitis	false negative, FN	true negative, TN

**Table 4:** Comparison of clinical characteristics and clinical laboratory data between patients with cystitis and patients with other cancers.

	Cystitis	Kidney Cancer	Prostate Cancer	Bladder Cancer	Uterus Cancer
	*n* = 144	*n* = 200	*n* = 201	*n* = 591	*n* = 200
age	60.12 ± 11.99	63.41 ± 10.45 **	71.83 ± 6.42 **	66.73 ± 9.4 **	60.86 ± 10.26
sex	88 (61.1%)	138 (69%)	201 (100%) **	386 (65.3%)	0 **
hypertension	34 (23.7%)	72 (36%) *	66 (32.8%)	173 (29.3%)	22 (11%) *
diabetes	20 (13.9%)	46 (23%) *	33 (16.4%)	93 (15.7%)	8 (4%) **
smoking	18 (12.5%)	51 (25.5%) *	47 (23.4%) *	138 (23.4%) *	9 (4.5%) *
drinking	23 (16%)	41 (20.5%)	64 (31.8%) **	118 (20%)	17 (8.5%) **
beetle nuts	2 (1.4%)	3 (1.5%)	3 (1.5%)	20 (3.4%)	1 (0.5%)
family history	1 (0.7%)	7 (3.5%)	5 (2.5%)	12 (2%)	7 (3.5%)
A/G Ratio	-	1.75 ± 0.4	1.07 ± 0.46	1.61 ± 0.47	1.64 ± 0.38
Albumin	3.96 ± 0.64	4.27 ± 0.57 **	4 ± 0.68	4 ± 0.68	4.15 ± 0.58 *
ALP	71 (55, 91)	66 (52, 79) **	66 (60, 72) **	69 (55, 90) **	65.5 (53, 79)
ALT	30.12 ± 42.66	31.46 ± 35.99	29.94 ± 32.49	27.45 ± 31.9	25.65 ± 28.02
AST	28.31 ± 32.07	30.56 ± 29.51	43.13 ± 74.83 *	40.65 ± 293.66	29.33 ± 53.46
BUN	14 (11, 21)	16 (12, 21) **	17 (13, 22.9) *	16 (12, 26) **	12 (9, 16.85) *
Calcium	9 (8.5, 9.4)	9.3 (8.85, 9.6)	8.895 (8.3, 9.4) **	9 (8.5, 9.4) **	9.2 (8.65, 9.65) **
Chloride	105.2 (103, 107.5)	105 (103, 107) *	105 (102.075, 107.225)	105 (102, 108) *	106 (104, 108) **
Creatinine	0.9 (0.7, 1.2)	1.1 (0.9, 1.5) **	1 (0.9, 1.2) *	1.1 (0.8, 1.5) **	0.7 (0.6, 0.8) **
Direct Bilirubin	0.11 (0.1, 0.2)	0.1 (0.1, 0.2) **	0.2 (0.1, 0.2)	0.1 (0.1, 0.2) *	0.1 (0.1, 0.18)
Estimated GFR	75.38 ± 33.22	62.68 ± 32.54 **	71.92 ± 27.16	64.13 ± 34 **	91.57 ± 28.86 **
Glucose AC	120.65 ± 47.39	123.11 ± 41.14	129.36 ± 56.6	124.83 ± 93.83	109.57 ± 26.12 *
pH	6.24 ± 0.89	6.03 ± 0.74 *	6.09 ± 0.85	6.26 ± 0.86	6.12 ± 0.71
Potassium	3.92 ± 0.5	4.14 ± 0.52 **	4.02 ± 0.48	4.12 ± 0.61 **	4.06 ± 0.46 **
Sodium	139 (137, 141)	139 (137, 140.775)	138 (136, 140) *	139 (137, 140.8)	139 (138.5, 141) **
Specific Gravity	1.016 (1.012, 1.021)	1.017 (1.013, 1.021)	1.017 (1.011, 1.022)	1.014 (1.01, 1.0195) *	1.016 (1.011, 1.021)
Total Bilirubin	0.9 ± 0.49	0.84 ± 0.31	1.08 ± 1.25	1.04 ± 1.93	0.81 ± 0.68
Total Cholesterol	190.15 ± 49.57	182.2 ± 42.36	189.88 ± 45.34	183.76 ± 44.58	200.1 ± 50.31
Total Protein	6.6 (6.0625, 7)	6.9 (6.55, 7.2) **	6.7 (6, 7.1)	6.7 (6.1, 7.1325) **	6.9 (6.3, 7.3)
Triglyceride	143.47 ± 88.07	141.88 ± 170.99	142.3 ± 89.64	131.97 ± 91.15	133.65 ± 141.94
Uric acid	5.82 ± 1.83	6.1 ± 1.64	6.36 ± 2.61	6.22 ± 1.88 *	5.4 ± 1.76
Urine epitheilum (UL)	-	0 (0, 6)	2.5 (0, 5.75)	3 (0, 9)	-
Urine epithelium count	0 (0, 2)	0 (0, 2)	0 (0, 2) **	1 (0, 3)	2 (0, 3) *
Nitrite		*p* = 0.001			
0	127 (88.2%)	194 (97%)	188 (93.5%)	493 (83.4%)	181 (90.5%)
1	17 (11.8%)	6 (3%)	13 (6.5%)	98 (16.6%)	19 (9.5%)
Strip WBC		*p* = 0.001	*p* = 0.003		
0	49 (34%)	140 (70%)	145 (72.1%)	298 (50.4%)	79 (39.5%)
1	24 (16.9%)	44 (22%)	34 (17%)	133 (62.1%)	79 (39.5%)
2	12 (8.3%)	12 (6%)	10 (5%)	73 (12.4%)	24 (12%)
3	13 (9%)	4 (2%)	12 (6%)	87 (14.7%)	18 (9%)
Urine Bilirubin					
0	137 (95.1%)	190 (95%)	190 (94.5%)	549 (94.9%)	194 (97%)
1	4 (2.8%)	10 (5%)	11 (5.5%)	28 (4.7%)	4 (2%)
2	2 (1.4%)	0	0	7 (1.2%)	2 (1%)
3	1 (0.7%)	0	0	7 (1.2%)	0
Urine Glucose					
0	86 (59.7%)	179 (89.5%)	183 (91%)	509 (86.1%)	185 (92.5%)
1	8 (5.6%)	12 (6%)	8 (4%)	52 (8.8%)	9 (4.5%)
2	1 (0.7%)	3 (1.5%)	2 (1%)	13 (2.2%)	3 (1.5%)
3	3 (2.1%)	6 (3%)	8 (4%)	17 (2.9%)	3 (1.5%)
Urine Ketone					
0	86 (59.7%)	184 (92%)	176 (87.6%)	523 (88.5%)	156 (78%)
1	10 (7%)	14 (7%)	24 (12%)	58 (9.8%)	38 (19%)
2	0	0	0	6 (1%)	5 (2.5%)
3	2 (1.4%)	2 (1%)	1 (0.5%)	4 (0.7%)	1 (0.5%)
Urine Protein					*p* < 0.0001
0	75 (52.1%)	124 (62%)	118 (58.7%)	279 (47.2%)	156 (78%)
Trace	16 (11.1%)	25 (12.5%)	25 (12.4%)	50 (8.5%)	15 (7.5%)
1	15 (10.4%)	19 (9.5%)	27 (13.4%)	91 (15.4%)	9 (4.5%)
2	24 (16.7%)	19 (9.5%)	23 (11.4%)	97 (16.4%)	12 (6%)
3	14 (9.7%)	13 (6.5%)	8 (4%)	74 (12.5%)	8 (4%)
Urobilinogen					*p* = 0.001
0	2 (1.4%)	7 (3.5%)	6 (3%)	20 (3.4%)	1 (0.5%)
0.1	55 (38.2%)	62 (31%)	59 (29.4%)	200 (33.8%)	124 (62%)
0.2	55 (38.2%)	84 (42%)	83 (41.3%)	261 (44.2%)	53 (26.5%)
1	29 (20.1%)	47 (23.5%)	52 (25.9%)	106 (17.9%)	20 (10%)
2	3 (2.1%)	0	1 (0.5%)	2 (0.3%)	1 (0.5%)
4	0	0	0	2 (0.3%)	1 (0.5%)
Urine occult Blood		*p* < 0.0001	*p* = 0.001		*p* = 0.016
0	52 (36.1%)	119 (59.5%)	112 (55.7%)	201 (34%)	85 (42.5%)
Trace	16 (11.1%)	23 (11.5%)	22 (10.9)	66 (11.2%)	26 (13%)
1	12 (8.3%)	25 (12.5%)	16 (8%)	53 (9%)	28 (14%)
2	19 (13.2%)	13 (6.5%)	21 (10.4%)	73 (12.4%)	29 (14.5%)
3	45 (31.3%)	20 (10%)	30 (14.9%)	198 (33.5%)	32 (16%)

* means *p* < 0.05, ** means *p* < 0.0001.

**Table 5:** The top selected feature from each comparison group.

Comparison Group		Top Selected Feature
cystitis	kidney cancer	Calcium
	prostate cancer	ALP
	bladder cancer	Albumin
	uterus cancer	Urine Ketone
kidney cancer	bladder cancer	Urine occult blood
	prostate cancer	ALP
	uterus cancer	Calcium
bladder cancer	prostate cancer	ALP
	uterus cancer	Creatinine

**Table 6:** Predictive performance of five models in discriminating patients with bladder cancer from patients with cystitis.

Models	Accuracy (95%CI)	Precision (95%CI)	f1 Score (95%CI)	Sensitivity (95%CI)	Specificity (95%CI)	AUC (95%CI)
decision tree	76.2% (71–81.5%)	77.9% (69.1–86.6%)	74.6% (69.8–79.5%)	73.2% (62.5–83.9%)	78.1% (64.9–91.3%)	0.775 (0.711–0.839)
random forest	83.1% (78.7–87.5%)	78.2% (71.5–85%)	81.6% (74.3–88.9%)	85.5% (76–94.9%)	79.4% (72–86.8%)	0.887 (0.826–0.947)
SVM	71.7% (63.5–80%)	81.9% (71.3–92.4%)	65.5% (51.9–79.1%)	55.7% (40.6–70.8%)	86.7% (78.9–94.5%)	0.736 (0.624–0.849)
XGBoost	82.8% (76.7–88.8%)	84.7% (74.5–94.9%)	82.7% (76.2–89.2%)	81.4% (75.1–87.7%)	83.3% (71–95.7%)	0.879 (0.819–0.939)
lightGBM	87.6% (81–94.1%)	86.3% (77.9–94.6%)	87.7% (81.8–93.5%)	89.5% (84.2–94.9%)	85.5% (75.2–95.7%)	0.932 (0.862–1.000)

**Table 7:** Predictive performance of the lightGBM model.

Groups	Accuracy (95%CI)	Precision (95%CI)	f1 Score (95%CI)	Sensitivity (95%CI)	Specificity (95%CI)	AUC (95%CI)
bladder cancer uterus cancer	86.9% (77.3–96.5%)	87.1% (76.5–97.7%)	87.3% (77.5–97%)	87.8% (77.3–98.3%)	86.7% (76.8–96.6%)	0.918 (0.849–0.988)
bladder cancer prostate cancer	84.8% (76–93.6%)	86.6% (76.8–96.4%)	85.1% (75.3–94.9%)	84.4% (71.9–96.9%)	85.1% (76.4–93.8%)	0.883 (0.823–0.942)
bladder cancer cystitis	87.6% (81–94.1%)	86.3% (77.9–94.6%)	87.7% (81.8–93.5%)	89.5% (84.2–94.9%)	85.5% (75.2–95.7%)	0.932 (0.862–1.001)
bladder cancer kidney cancer	84.5% (78.3–90.6%)	83% (73.2–92.9%)	84.5% (78.3–90.7%)	86.8% (79.9–93.7%)	82.9% (72.7–93.2%)	0.928 (0.88–0.977)
kidney cancer cystitis	86.2% (78.2–94.2%)	86.8% (78–95.6%)	86.9% (79–94.9%)	88% (76.5–99.6%)	84.2% (73–95.5%)	0.903 (0.854–0.952)
uterus cancer cystitis	83.8% (77.1–90.5%)	87% (76.8–97.3%)	83.7% (76.7–90.7%)	81.8% (71.2–92.5%)	86.5% (76.9–96.1%)	0.915 (0.834–0.995)
prostate cancer cystitis	87.6% (82.9–92.2%)	88.5% (80.3–96.6%)	87% (81.7–92.4%)	86.7% (77.5–95.8%)	88.4% (77.9–98.9%)	0.944 (0.903–0.986)
prostate cancer uterus cancer	84.1% (77–91.3%)	82.5% (68.2–96.7%)	82.3% (70.1–94.5%)	82.7% (70.5–94.9%)	84.7% (76.4–93.1%)	0.897 (0.837–0.957)
kidney cancer uterus cancer	86.2% (78.4–94%)	87.1% (76.5–97.7%)	86.8% (79–94.5%)	87.1% (79.5–94.6%)	86.2% (75.6–96.9%)	0.923 (0.858–0.988)
kidney cancer prostate cancer	84.5% (77.7–91.2%)	82.1% (72.7–91.6%)	84.9% (79.1–90.7%)	88.6% (82.5–94.6%)	81.3% (69.1–93.5%)	0.91 (0.849–0.972)

## Data Availability

The data was placed in the supplementary material and GitHub (https://github.com/I-JUNG-TSAI/Predict-BC-Clinical-Laboratory-Data, accessed on 14 June 2021).

## Supplementary Materials

The following supporting information can be downloaded at: https://www.mdpi.com/article/10.3390/diagnostics12010203/s1, Table S1: Comparison of predictive performance with no sampling, oversampling, and undersampling.
